# The Natural Combination Medicine Traumeel (Tr14) Improves Resolution of Inflammation by Promoting the Biosynthesis of Specialized Pro-Resolving Mediators

**DOI:** 10.3390/ph14111123

**Published:** 2021-11-03

**Authors:** Paul M. Jordan, Emeline van Goethem, Andrea M. Müller, Kathrin Hemmer, Virginie Gavioli, Vincent Baillif, Yvonne Burmeister, Natascha Krömmelbein, Marc Dubourdeau, Bernd Seilheimer, Oliver Werz

**Affiliations:** 1 Department of Pharmaceutical/Medicinal Chemistry, Institute of Pharmacy, Friedrich-Schiller-University Jena, Philosophenweg 14, 07743 Jena, Germany; paul.jordan@uni-jena.de; 2 Ambiotis SAS, 3 Rue des Satellites, 31400 Toulouse, France; emeline.vangoethem@ambiotis.com (E.v.G.); virginie.gavioli@ambiotis.com (V.G.); Vincent.Baillif@ambiotis.com (V.B.); marc.dubourdeau@ambiotis.com (M.D.); 3 Heel GmbH, Dr. Reckeweg-Str. 2-4, 76532 Baden-Baden, Germany; andrea.mueller@heel.com (A.M.M.); Kathrin.hemmer@heel.com (K.H.); YvonneBurmeister@gmx.de (Y.B.); nkroemmelbein@googlemail.com (N.K.); bernd.seilheimer@heel.com (B.S.)

**Keywords:** lipid mediator, specialized pro-resolving mediators, resolution index, inflammation, efferocytosis, peritonitis, Traumeel, multicomponent, inflammation resolution

## Abstract

The resolution of inflammation is an integral part of the acute inflammatory response and eventually leads to the return to homeostasis. It is supported by specialized pro-resolving mediators (SPMs) that act as immunoresolvents via specific G-protein-coupled receptors. In contrast to classical non-steroidal anti-inflammatory drugs (NSAIDs) that suppress the formation of pro-inflammatory lipid mediators such as prostaglandins, novel pharmacotherapeutic concepts propose to foster the biosynthesis of beneficial SPMs. Here, we demonstrate that the natural combination medicine Traumeel (Tr14) improves resolution of inflammation by promoting SPM formation. Tr14 enhanced the biosynthesis of 12-/15-lipoxygenase (LOX) products and of SPMs in zymosan-induced mouse peritonitis as well as in human monocyte-derived macrophages challenged with *Staphylococcus aureus*. Importantly, in the peritonitis model, Tr14 supported the recruitment of innate leukocytes and the efferocytotic capacity of macrophages, and positively influenced the inflammation resolution index. Taken together, we suggest that based on these properties Tr14 may possess therapeutic potential as an enhancer for the resolution of inflammatory processes.

## 1. Introduction

Non-steroidal anti-inflammatory drugs (NSAIDs) are amongst the most popular medicines and are widely used as analgesics and for reducing inflammation [1]. Despite their efficacy in alleviating pain and inflammatory reactions, NSAIDs exert adverse gastrointestinal, cardiovascular, and renal effects [2], and they are essentially inefficient at promoting resolution and tissue repair [3]. Acute inflammation is usually self-limited and terminated in a temporal manner governing the return to tissue homeostasis [4,5]. The resolution of inflammation is part of the inflammatory process that actively terminates inflammation and leads to tissue repair and regeneration [6]. It is characterized by normalization of chemokine gradients and by the clearance of apoptotic neutrophils by macrophages [7,8,9,10]. In the early stages of acute inflammation, pro-inflammatory lipid mediators (LMs) are generated by resident cells in injured tissue which elicit recruitment of polymorphonuclear leukocytes (PMNs) that further enhance pro-inflammatory mediator production [6,11,12,13]. The pro-inflammatory LMs are comprised of leukotrienes (LTs) and prostaglandins (PGs) produced from arachidonic acid (AA) by the initial actions of the key enzymes 5-lipoxygenase (5-LOX) and cyclooxygenases (COX), respectively [13]. The biosynthesis of PGs is blocked by NSAIDs that suppress the cardinal signs of inflammation, namely, redness, heat, swelling, and pain [1,13,14]. In contrast to pro-inflammatory LMs, the specialized pro-resolving mediators (SPMs) such as lipoxins (LXs), resolvins (RVs), maresins (MaRs) or protectins (PDs) generated from arachidonic acid (AA; LXs), eicosapentaenoic acid (EPA; RVs E-series) and docosahexaenoic acid (DHA; RVs D-series, MaRs, PDs) coordinate the subsequent resolution phase [6,10]. SPMs govern resolution of inflammation via defined G-protein-coupled receptors (GPCRs) and accelerate tissue repair and regeneration [15,16]. They limit excessive neutrophil infiltration, promote the sequestering of pro-inflammatory cytokines and the efferocytotic actions of macrophages, which accomplishes resolution of inflammation without immunosuppression [17,18]. Therefore, SPMs are considered as valuable immunoresolvents that constitute alternatives to NSAIDs for pharmacotherapy of inflammatory diseases.

Here we investigated the natural combination medicine Traumeel (Tr14) for its impact on the resolution phase of acute inflammation. Earlier studies showed that Tr14 significantly influences inflammatory reactions. For example, Tr14 impaired induced hind paw edema and decreased interleukin (IL)-6 formation in a rat model of blood-induced inflammation [19]. In a wound healing mouse model, Tr14 differentially expressed genes related to key wound repair pathways, such as cellular differentiation, wound contraction, and cell mobility [20]. The antioxidant capability of Tr14 inhibited the oxidative burst of peripheral neutrophils in patients with periodontitis [21]. Tr14 also diminished the detrimental effects of excessive chronic noise on microvascular integrity in rats apparently through mast cell stabilization properties [22]. Importantly, also in clinical trials, Tr14 affected cytokine levels in randomized, double-blind controlled trials (RCTs) of exercise-induced muscle trauma [23,24]. 

In this study, we used the zymosan-induced murine peritonitis model to assess the capacity of Tr14 to promote inflammation resolution. Zymosan-induced peritonitis represents an acute local inflammation with a peak in leukocyte recruitment after 4 to 12 h [15] and subsequent resolution phase after 12 to 24 h [16]. We studied whether Tr14 affects leukocyte recruitment, LM profiles and relevant genes for LM-biosynthetic enzymes in peritoneal exudates of treated mice. Moreover, we analyzed the effects of Tr14 on LM pathways in human monocyte-derived macrophages (MDMs) with M1- and M2-like phenotypic properties. Our results show that Tr14 acts as an enhancer for the resolution phase of acute inflammation along with elevating SPM levels.

## 2. Results

### 2.1. Tr14 Affects Lipid Mediator Pathways and Promotes Resolution of Inflammation in Murine Peritonitis

**Figure 1:**
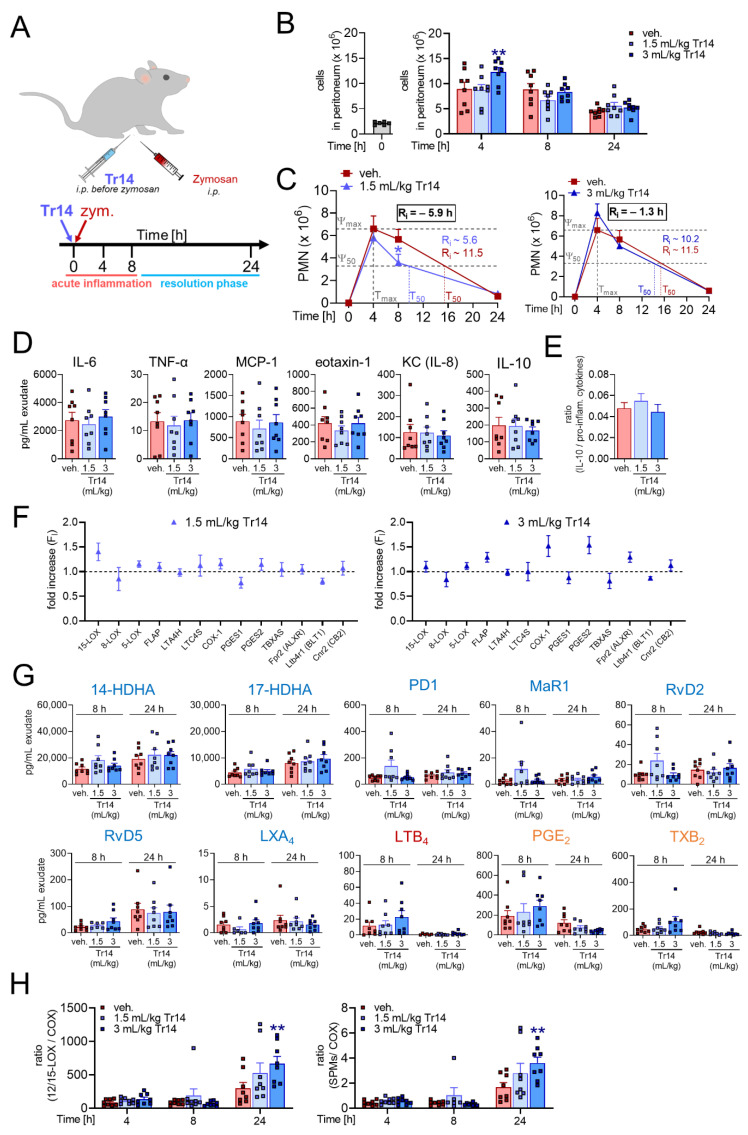
Effects of Tr14, pre-administered *i.p.* over 6 days prior murine peritonitis induction, on resolution of inflammation and on lipid mediator pathways. (**A–H**) Self-resolving inflammation was initiated by injection of zymosan (0.1 mg/mouse, *i.p.*) into mice. Before zymosan injection, Tr14 (1.5 mL/kg or 3 mL/kg) or vehicle (veh., 0.9% NaCl) were administered *i.p.* once daily for six days. Peritoneal exudates were collected after 4, 8 and 24 h (*n* = 6–8; outliers were removed) post-zymosan injection, and from naïve untreated mice representing time point 0 h (*n* = 6). (**A**) Scheme of administration of Tr14 and induction of peritonitis. (**B**) Cell numbers in the peritoneum, shown as single values and mean + SEM for the indicated time points. Left panel shows cell numbers in the peritoneum of naïve (untreated) mice at t = 0 h. ** *p* < 0.01; *p* values were calculated versus vehicle for each time point; unpaired two-way ANOVA with Dunnett’s multiple comparison test. (**C**) Numbers of infiltrated PMNs (GR1^+^, F4/80^−^) in the peritoneal lavages, shown as mean + SEM at the indicated time points. Resolution indices were defined, including Ψ_max_ (maximal PMN counts), T_max_ (time point when PMNs reach Ψ_max_), Ψ_50_ (50% PMN number reduction), T_50_ (time point corresponding to Ψ_50_) and R_i_ (resolution interval, the interval between T_max_ and T_50_) according to [26], * *p* < 0.01; *p* values were calculated versus vehicle for each time point; unpaired two-way ANOVA with Dunnett’s multiple comparison test. (**D**) Cytokine/chemokine levels were measured 4 h post-zymosan injection, shown in pg/mL exudate as single values and mean + SEM. (**E**) Ratio of anti-inflammatory IL-10 versus pro-inflammatory cytokines/chemokines (sum of IL-6, TNF-α, MCP-1, eotaxin-1 plus KC (IL-8)) after 4 h of zymosan injection. (**F**) mRNA levels of LM-biosynthetic enzymes and of LM receptor-related genes, analyzed by RT-PCR 4 h post-zymosan injection. Data are given as mean ± SEM as fold increase of vehicle group. (**G**) LM levels in peritoneal lavages after 8 and 24 h were analyzed by UPLC-MS-MS and are shown in pg/mL exudate as single values and as mean + SEM as bar chart. (**H**) Ratio of 12/15-LOX products (17-HDHA and 14-HDHA; left panel) and SPMs (LXA_4_, PD1, MaR1, RvD2 and RvD5; right panel) versus COX products (PGE_2_ and TXB_2_) after 4, 8 and 24 h of zymosan injection. Results are shown as single values and mean + SEM; ** *p* < 0.01; *p* values were calculated versus vehicle for each time point; unpaired two-way ANOVA with Dunnett´s multiple comparison test.

**Figure 2:**
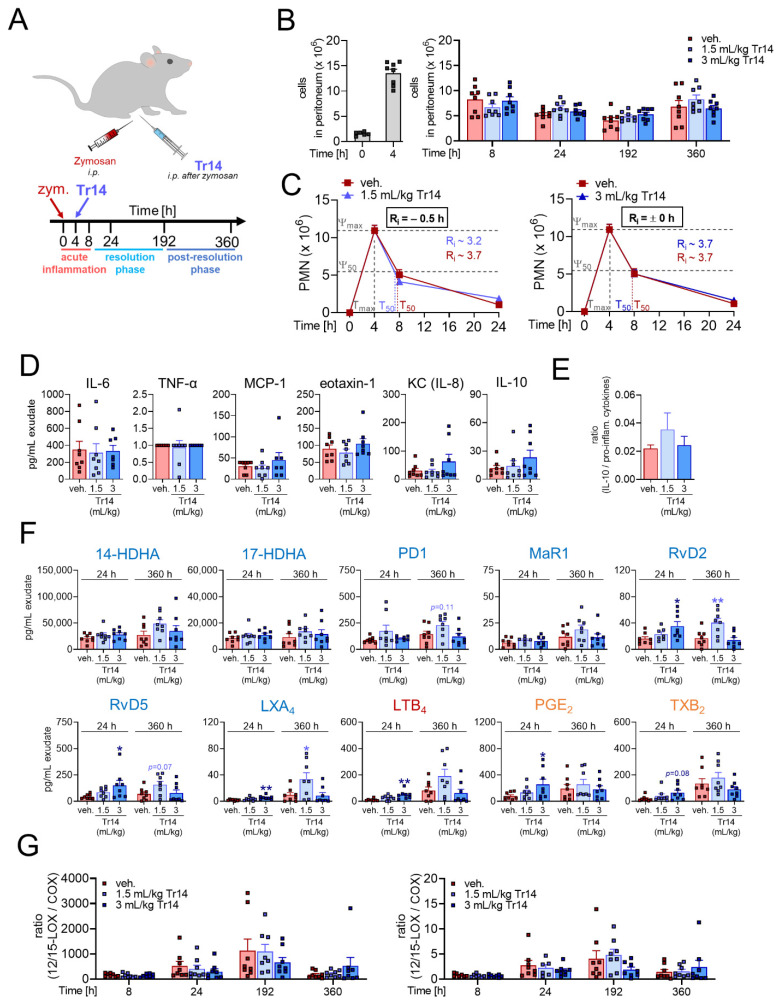
Effects of Tr14, administered *i.p.* 4 and 8 h post-induction of murine peritonitis, on resolution of inflammation and on lipid mediator pathways. (**A–G**) Self-resolving inflammation was initiated by injecting zymosan (0.1 mg/mouse, *i.p.*) into mice. Tr14 (1.5 mL/kg or 3 mL/kg) or vehicle (veh., 0.9% NaCl) were administered *i.p.* 4 and 8 h post-zymosan injection. Peritoneal exudates were collected after 4, 8, 24, 192 and 360 h (*n* = 6–8; outliers were removed) after zymosan injection, and from naïve untreated mice representing time point 0 h (*n* = 6). (**A**) Scheme of administration of Tr14 and induction of peritonitis. (**B**) Quantification of cell numbers in the peritoneal lavages, shown as single values and mean + SEM for the indicated time points. Left panel shows cell numbers in the peritoneum of naïve (untreated) mice at t = 0 and of zymosan-treated mice at t = 4 h. (**C**) Numbers of infiltrated PMNs (GR1^+^, F4/80^−^) in the peritoneal lavages, shown as mean + SEM at indicated time points. Resolution indices were defined, including Ψ_max_ (maximal PMN counts), T_max_ (time point when PMNs reach Ψ_max_), Ψ_50_ (50% PMN number reduction) T_50_ (time point corresponding to Ψ_50_) and R_i_ (resolution interval, the interval between T_max_ and T_50_) according to [26]. (**D**) Cytokine/chemokine levels were measured 8 h post-zymosan injection and are given in pg/mL exudate as single values and mean + SEM. (**E**) Ratio of anti-inflammatory IL-10 versus pro-inflammatory cytokines/chemokines (sum of IL-6, TNF-α, MCP-1, eotaxin-1 plus KC (IL-8)) after 8 h of zymosan injection. (**F**) LM levels in exudates 24 and 360 h post-zymosan injection were analyzed by UPLC-MS-MS and are given in pg/mL exudate as single values and mean + SEM, * *p* < 0.05; ** *p* < 0.01; *p* values were calculated versus vehicle for each time point; unpaired one-way ANOVA with Dunnett´s multiple comparisons test. (**G**) Ratio of 12/15-LOX products (17-HDHA and 14-HDHA; left panel) and SPMs (LXA_4_, PD1, MaR1, RvD2 and RvD5; right panel) versus COX products (PGE_2_ and TXB_2_) 4, 8, 24, 192 and 360 h after zymosan injection. Results are shown as single values and mean + SEM.

To study the effects of Tr14 on the inflammation-resolving mechanism *in vivo*, we employed the well-characterized acute inflammation model of zymosan-induced peritonitis in mice. Two different experimental settings were applied (see Section 4 and Figure S1 for more details): (1) Tr14 was given intraperitoneally (i.p.) at low (1.5 mL/kg) or high dose (3 mL/kg) once daily for six days prior to zymosan (i.p.) injection and peritoneal exudates were collected after 4, 8 and 24 h (Figure 1A); (2) Tr14 was given i.p. at low (1.5 mL/kg) or high dose (3 mL/kg) 4 and 8 h post-zymosan (i.p.) injection and peritoneal exudates were collected after 4, 8, 24, 192 and 360 h (Figure 2A).

In the experimental setting (1), where Tr14 was given before zymosan, the maximum cell infiltration was reached at 4 h post-zymosan (i.p.) injection. Tr14 at the high dose significantly increased the total cell number in the peritoneum after 4 h (Figure 1B). The infiltrate was predominantly composed of PMNs that were significantly decreased in numbers by the low dose of Tr14 at 8 h (Figure 1C, Figure S2A). Additionally, Tr14 significantly enhanced the recruitment of monocytes/macrophages after 4 and 24 h and significantly increased eosinophil recruitment after 4 h (Figure S2A,B). Tr14 shortened the resolution interval (R_i_—interval between T_max_ and T_50_) with the high dose by 1.3 h and with the low dose by 5.9 h (Figure 1C). Tr14 did not alter the release of IL-6, TNF-α, monocyte chemoattractant protein-1 (MCP-1), eotaxin-1, KC (IL-8) or IL-10 after 4 h (Figure 1D and Figure S2C) and did not affect the ratio of anti-inflammatory IL-10 versus the sum of the pro-inflammatory cytokines/chemokines IL-6, TNF-α, MCP-1, eotaxin-1, and KC (Figure 1E). We then investigated the effect of Tr14 on the expression of 13 genes related to LM-biosynthetic enzymes or LM receptors, and of other 17 inflammation-related genes (mainly cytokines) in the peritoneal lavage. After 4 h, Tr14 at high and low dose slightly increased the mRNA levels of 15-LOX (Figure 1F), IL-2, IL-4, and IL-15 (Figure S2D), which are involved in anti-inflammatory signaling. Furthermore, Tr14 decreased mRNA levels of pro-inflammatory cytokines such as IL-5 and IL-17A (Figure S2D). Next, we assessed the levels of LM in the peritoneal exudates using UPLC-MS-MS [25]. In the absence of Tr14, the pro-inflammatory COX-derived PGE_2_ and TXB_2_ along with 5-LOX-derived LTB_4_ were mainly produced 4 to 8 h post-zymosan and their levels were diminished after 24 h (Figure 1G and Figure S2E). In contrast, the levels of monohydroxylated SPM precursors (i.e., 14-HDHA and 17-HDHA) and of various SPMs (i.e., PD1, MaR1, RvD2, RvD5 and LXA_4_) were continuously formed from 8 h up to 24 h (Figure 1G and Figure S2E). Pre-treatment with Tr14 prior zymosan did not markedly affect the overall production of LMs, although a tendency of decreased COX products (PGE_2_ and TXB_2_) was observed after 24 h (Figure 1G and Figure S2E). However, Tr14 significantly increased the ratio of 12/15-LOX-derived products (14-HDHA and 17-HDHA) and SPMs (PD1, MaR1, RvD2, RvD5 and LXA_4_) versus COX-derived (PGE_2_ and TXB_2_) LMs implying that Tr14 promotes a switch from pro-inflammatory towards pro-resolving LMs (Figure 1H).

When Tr14 was administered after zymosan injection (experimental setting (2), Figure 2A), no significant change in cell counts was observed after 24 h versus vehicle group (Figure 2B and Figure S3A). However, for the post-resolution phase (192 h up to 360 h after zymosan), Tr14 at low dose significantly increased numbers of resident macrophages and lymphocytes (Figure S3A). In comparison to vehicle control, the cell-type composition remained unaltered after 24 h of Tr14 treatment (Figure S3B) but slightly changed to more lymphocytes and resident macrophages after low-dose Tr14 treatment after 360 h (Figure S3B). Note that Tr14 at both doses did not affect the resolution indices (Figure 2C). Released cytokines in the exudates peaked 4 h post-zymosan administration and were barely present at other timepoints. Tr14 weakly influenced the release of cytokines (Figure S3C) with a tendency towards elevated amounts of anti-inflammatory IL-10 and chemokine KC (IL-8) (Figure 2D) and towards an increase of the ratio of anti-inflammatory IL-10 versus the sum of the pro-inflammatory cytokines/chemokines IL-6, TNF-α, MCP-1, eotaxin-1, and KC after 8 h post-zymosan administration (Figure 2E). In the absence of Tr14, SPMs and especially 12/15-LOX products (14-HDHA and 17-HDHA) as well as the 5-LOX product LTB_4_ were increasingly produced up to 360 h (Figure 2F and Figure S3D). Strikingly, Tr14, especially at the low dose, significantly increased the levels of SPMs such as RvD2, RvD5 and LXA_4_ after 24 h as well as for the post-resolution time (360 h) against vehicle (Figure 2F and Figure S3D). In comparison to experimental setting (1), application of Tr14 after zymosan injection did not significantly alter the ratio of 12/15-LOX-derived products (14-HDHA and 17-HDHA) and SPMs (PD1, MaR1, RvD2, RvD5 and LXA_4_) versus COX-derived (PGE_2_ and TXB_2_) LMs (Figure 2G).

### 2.2. Tr14 Enhances Macrophage Efferocytotic Actions In Vivo

**Figure 3:**
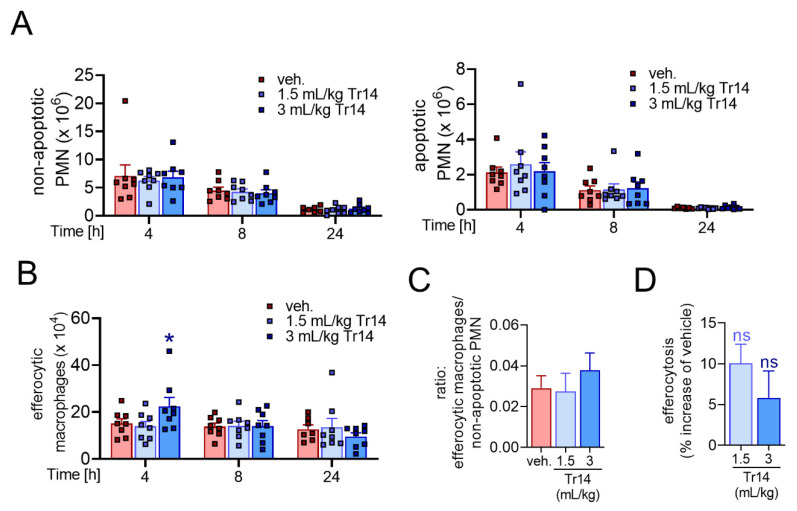
Tr14 enhances macrophage efferocytotic actions in murine peritonitis. (**A**–**D**) Self-resolving inflammation was initiated by injection of zymosan (0.1 mg/mouse, i.p.) into mice. Prior to zymosan injection, Tr14 (1.5 mL/kg or 3 mL/kg) or vehicle (veh., NaCl 0.9%) were administered i.p. once daily for six days. Peritoneal exudates were collected 4, 8 and 24 h post-zymosan injection (*n* = 8), and from naïve untreated mice representing time point 0 h (*n* = 6). (**A**) Infiltrated PMNs (Ly6G^+^, F4/80^−^) were stained with annexin-V and DAPI to determine non-apoptotic PMNs (Ly6G^+^, F4/80^−^, annexin-V^−^, DAPI^−^, left) and early and late apoptotic PMNs (Ly6G^+^, F4/80^−^, annexin-V^+^, DAPI^−/+^, right) in the peritoneal lavages, shown as single values and mean + SEM at indicated time points. (**B**) Numbers of efferocytotic macrophages (F4/80^+^, Ly6G^+^) in the peritoneal lavages, given as single values mean + SEM at indicated time points. * *p* < 0.05, versus vehicle; two-way ANOVA with Dunnett´s multiple comparisons test. (**C**) Ratio of efferocytotic macrophages versus non-apoptotic PMN cell counts 4 h post-zymosan injection. Results are shown as mean + SEM. (**D**) Mean fluorescent intensity (MFI) of Ly6G^+^ PMNs in efferocytotic macrophages (F4/80^+^) after 4 h, measured by flow cytometry, given as % increase versus vehicle. Results are shown as mean + SEM.

Efferocytosis of apoptotic cells and cellular debris by recruited macrophages is an essential step in the resolution process that is strongly promoted by SPMs [6]. We investigated the effect of Tr14 administered i.p. once daily for six days prior to zymosan injection (experimental setting (1)) on macrophage efferocytotic functions in the murine peritonitis model (Figure S1). First, we assessed the total numbers of non-apoptotic and apoptotic PMNs after 4, 8 and 24 h in the exudates. Tr14 did not significantly change the numbers of apoptotic and non-apoptotic PMNs (Figure 3A). Next, we assessed the number of efferocytotic macrophages. Interestingly, Tr14 significantly enhanced the number of efferocytotic macrophages after 4 h (Figure 3B). The ratio of efferocytotic macrophages versus non-apoptotic PMNs was higher in Tr14-pre-treated mice after 4 h, which suggests acceleration of inflammation resolution (Figure 3C). Moreover, mice pretreated with Tr14 revealed a tendency towards enhanced capacity for PMN uptake by each efferocytotic macrophage versus cells from vehicle-treated animals (Figure 3D). Together, our data show that Tr14 enhances the formation of SPM and, arguably as a consequence, promotes the efferocytosis of apoptotic PMNs by macrophages *in vivo* during peritonitis in mice.

### 2.3. Tr14 Increases 15-Lox-1-Mediated Lipid Mediator Biosynthesis in Human M2 Macrophages

**Figure 4:**
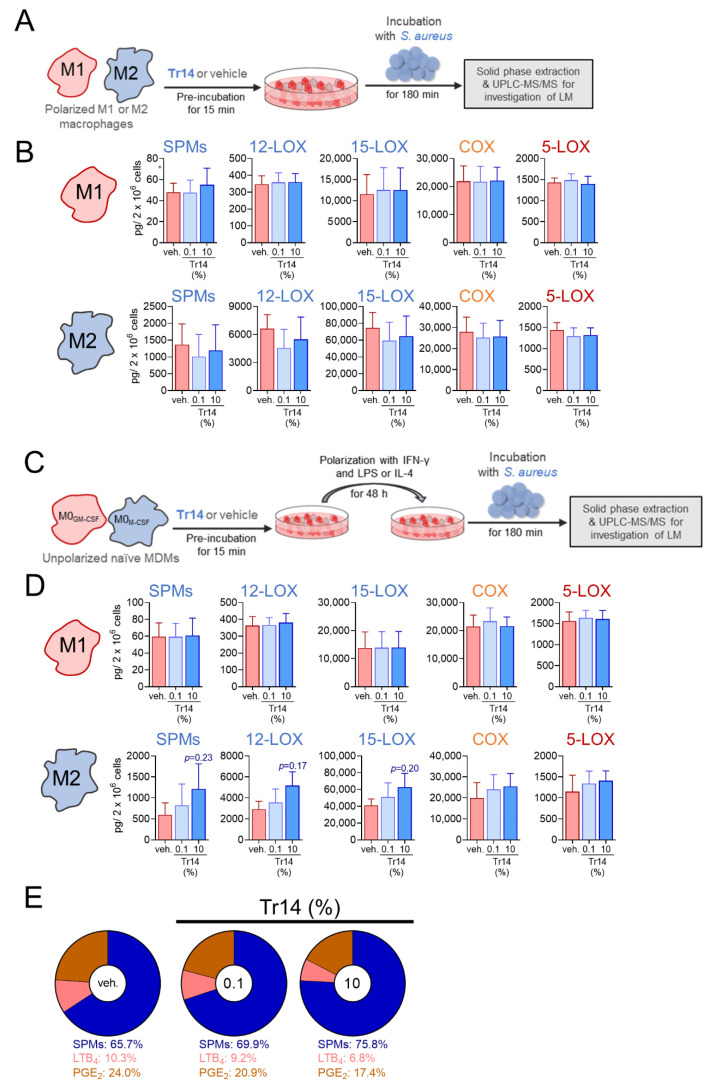
High dose Tr14 increases 15-LOX-1-mediated lipid mediator biosynthesis in human M2 MDM. (**A**) Scheme of administration of Tr14 to polarized macrophages prior incubation with *S. aureus*. (**B**) Human M1- and M2-MDM (2 × 10^6^ cells/mL) were preincubated in PBS pH 7.4 containing 1 mM CaCl_2_ with 0.1 or 10% of Tr14 or vehicle (veh., 0.9% NaCl solution) for 15 min at 37 °C and then incubated with *S. aureus* (LS1; ratio 1:50) for another 180 min. Formed LM were extracted from the supernatants and analyzed by UPLC-MS-MS and are given as means + SEM; *n* = 3 separate donors. (**C**) Scheme of administration of Tr14 to macrophages prior to polarization. (**D**) Human macrophages (M0) were preincubated with 0.1 or 10% of Tr14 or vehicle (0.9% NaCl solution) for 15 min and then polarized for 48 h with LPS/IFN-γ to M1- or with IL-4 towards M2-MDM. Then, cells were incubated with *S. aureus* (LS1; ratio 1:50) in PBS pH 7.4 containing 1 mM CaCl_2_ for another 180 min. Formed LM were extracted from the supernatants and analyzed by UPLC-MS-MS and are given as means + SEM; *n* = 3 separate donors. Statistical analysis was performed using matched one-way ANOVA with Dunnett´s multiple comparison test; relevant *p* values are given in the figure. (**E**) Amounts of PGE_2_ (orange), LTB_4_ (red) and SPMs (blue) formed in the M2-MDM that had been treated with vehicle, 0.1 or 10% Tr14 prior polarization (panel **D**) are shown in pie charts; data are given as %, *n* = 3.

To investigate the effect of Tr14 on the biosynthesis of 5-LOX-, COX-, and 12/15-LOX-derived LM biosynthesis *in vitro* in human innate immune cells, we preincubated MDM that had been polarized towards M1 and M2 phenotypes for 48 h, with Tr14 (0.1 or 10%) for 15 min and exposed them to *S. aureus* (MOI = 1:50) as stimulus to elicit LM formation. Previous studies showed that *S. aureus* elicits the formation of a broad spectrum of LM in such human MDM, where the M1 phenotype mainly generates pro-inflammatory PGs and LTs, and M2 are a major source for SPMs and 15-LOX-derived precursors [27]. First, we excluded that Tr14 possesses cytotoxic effects on MDMs over an incubation time of 48 h (Figure S4A). Tr14 did not significantly impact the LM biosynthesis in short-term incubations (up to 3 h, Figure 4A,B), different from classical NSAIDs ibuprofen and diclofenac, the 5-LOX inhibitor zileuton, and the specific 15-LOX-1 inhibitor BLX-3887 [28].

In contrast, when naïve MDMs were pre-treated with Tr14 for 15 min, then polarized towards M1- or M2-like phenotypes for 48 h, and subsequently challenged by *S. aureus* for LM formation, an enhanced formation of 15-LOX-1-mediated LM, especially SPMs, was observed in M2-MDM (Figure 4C,D and Figure S4B). As a result, Tr14 diminished the proportion of produced LTB_4_ and PGE_2_ versus SPMs (Figure 4E) suggesting that Tr14 shifts LM formation in M2-MDM towards a more pro-resolution profile during polarization of macrophages.

## 3. Discussion

NSAIDs are used for pain and inflammation management with decades of documented success. However, they are also well-known for their gastric, cardiovascular, renal, hepatic, and hematologic side effects [29]. It is suggested that, among many reasons, LM biosynthesis shunting phenomena and impairment of SPM formation are accounting for these side effects [28]. However, if SPM formation and thus resolution signaling is impaired, pro-inflammatory signals may accumulate, leading to excessive inflammation and, subsequently, to disease [17,18]. A frequently used approach to reduce the severity and number of side effects of NSAIDs is the use of respective prodrugs of the active substances that especially limit gastric toxicity [30]. Alternative strategies to NSAIDs could be smart manipulation of the overall LM networks to stimulate signaling in resolution pathways. In this respect, SPMs as novel immunoresolvents have been investigated in numerous experimental studies and showed promising therapeutic potential for treatment of inflammatory diseases [6,7,18,31].

The objective of this study was to investigate the potential pro-resolution properties of Tr14. We show that Tr14, which has been studied in various experimental models and clinical trials for its effect on inflammatory conditions [19,20,21,22,23,24], has a favorable impact on the inflammation resolution processes in mice along with a significant ability to elevate SPM levels. We found that in a mouse model of self-resolving inflammation triggered by zymosan *in vivo,* Tr14 did not consistently inhibit pro-inflammatory actions, such as recruitment of PMNs, release of cytokines or production of LTs and PGs, which contrasts with classical NSAIDs [1]. In this respect, Tr14 should not elicit the typical side effects of NSAIDs that essentially act as COX inhibitors. Our data demonstrate that Tr14 rather promotes the resolution process of inflammation, reflected by shortened resolution intervals by pre-treatment with Tr14 and elevated numbers of efferocytotic macrophages important for clearance of apoptotic PMNs and cellular debris produced during the onset of inflammation [32]. These macrophage actions are typically stimulated by SPMs such as MaR1 [33], and our data show that Tr14 shifts the LM profiles towards SPMs in the mouse peritonitis model *in vivo* as well as in human macrophages on the cellular level. Taken together, Tr14 positively influenced several pro-resolution properties, that is, it shortened the resolution interval, enhanced macrophage efferocytosis and increased SPM levels.

Our findings reveal that Tr14 directly targets cells of the innate immune system supported by the stimulatory effect on SPM formation in human M2-MDM. We recently showed that, in contrast to M1-MDM, the M2 phenotype produces substantial amounts of SPMs upon exposure to pathogenic bacteria [27] that release exotoxins such as HLA to elicit 15-LOX-1 activation and concomitant SPM formation involving ADAM10 [34]. The capacity to produce SPMs and their 15-LOX-derived precursors by M2-MDM was increased when Tr14 was present during macrophage polarization. Note that short-term pre-treatment of polarized M2-MDM with Tr14 failed to increase 15-LOX-derived products. This would exclude direct stimulatory effects of Tr14 on the LM-biosynthetic machinery and signaling such as Ca^2+^ mobilization, 15-LOX translocation or supply of fatty acid substrates (i.e., AA, EPA or DHA) [34]. Possibly, Tr14 increases 15-LOX-1 or 15-LOX-2 protein levels, but priming of the M2-MDMs for more sustained Ca^2+^ mobilization is also reasonable. Along these lines, we previously reported that besides the expression of 15-LOXs the activation on 15-LOX-1 depends on a slow and sustained Ca^2+^-influx into M2-MDMs [27].

Our data showing that Tr14 increased SPM production, support previous clinical reports about Tr14′s analgesic action. Indeed, SPMs were shown to reduce pain [35] and in the management of osteoarthritis of the knee, Tr14 co-administered with Zeel (Ze14), another natural combination medicine, reduced moderate-to-severe pain in the affected knee [36]. Since Tr14 enhanced efferocytotic actions of macrophages in mice, increased SPM levels and promoted inflammation resolution (shortening the resolution interval), we suggest that Tr14 may diminish pain in arthritic diseases by promoting the clearance of damage-associated molecular patterns (DAMPs) that are major causes for activated nociceptive neurons in the osteoarthritic joints [37]. This is in line with the reported effect of topical Traumeel application that successfully reduced mild-to-moderate post-injury pain and improved ankle mobility as effectively as 1% diclofenac gel [38].

## 4. Materials and Methods

### 4.1. Interventions

Traumeel (Tr14) was obtained free of charge from Heel GmbH (Baden-Baden, Germany). Tr14 was prepared in accordance with GMP standards and supplied in glass ampoules prepared for injection. The active ingredients of Tr14 are listed in Table S1. Each 1.1 mL ampoule of the vehicle control contained 0.9% sodium chloride for injection.

### 4.2. Animals, Animal Care and the Ethical Statement

Male C57BL/6J mice (7–12 weeks of age) with a weight range from 21 to 26 g, obtained from Charles River (Écully, France), were housed in a controlled environment (21 ± 2 °C) and provided with standard rodent chow and water. Animals were allowed to acclimate for seven days prior to experiments and were subjected to 12 h light/12 h dark schedule. Experiments were conducted during the light phase. The experimental procedures were approved ethically by the Comité d’Ethique en Expérimentation Animale (CEEA) 122 with the protocol number of 22923-201911251226765v3.

### 4.3. Study Design

The *in vivo* experiment was a randomized, three-arm parallel, vehicle-controlled exploratory study. Three intervention conditions were defined for each experimental setting: vehicle, Tr14 “high-dose”, and Tr14 “low-dose”. Two different experimental settings were applied: (1) preventive pre-treatment injection, when the intervention was administered intraperitoneally (*i.p.*) before induction of inflammatory response and peritoneal exudates were collected after 4, 8 and 24 h; (2) curative post-induction injection, when the intervention was administered *i.p.* after induction of inflammatory response and peritoneal exudates were collected after 4, 8, 24, 192 and 360 h (Figure S1). The experimental baseline was expressed as time point 0 h and resulted from the samples of a separate group of 6 naïve untreated animals.

For each time point and each intervention condition, one group of 8 mice was used. Each animal was considered as an experimental unit (Figure S1). Mice were assigned for the experiments according to their body weights.

The parameters assessed were inflammatory cell trafficking (flow cytometry), resolution indices (magnitude, duration, resolution interval; described in [26]), LM profiling, production of cytokines, gene expression in pro-inflammatory and pro-resolution pathways, PMN apoptosis (flow cytometry), and efferocytosis (flow cytometry).

For the *in vitro* studies peripheral blood mononuclear cells (PBMC) isolated from three healthy volunteers were used and differentiated to macrophages. These monocyte-derived macrophages (MDM) were subjected to polarization towards M1 or M2 phenotypes and incubated with *Staphylococcus aureus* for induction of LM formation. Three intervention conditions were defined: Tr14 (low concentration = 0.1%) or Tr14 (high concentration = 10%) and vehicle with three independent biological replications (different donors). Experiments were performed in singlet technical measurements with cells from *n* = 3 different donors at different days, according to experiences and results from previous studies [27,34]. To explore whether and how Tr14 affects LM biosynthesis in M1- and M2-MDMs two experimental settings were applied: (1) polarized M1- and M2-MDMs were treated with the test item after polarization to investigate an effect on LM-producing enzyme activation; (2) MDMs were pre-treated with the test item prior to polarization to investigate the impact on LM-producing enzymes production. The assessed parameters were cell viability (MTT assay), and LM profiling (metabololipidomics).

### 4.4. Zymosan-Induced Murine Peritonitis and Peritoneal Lavage Collection

Zymosan (0.1 mg/mouse; InvivoGen, Toulouse, France) was injected into the peritoneum of mice for all tested groups, except for the baseline group (time point 0 h). Tr14 at 3 mL/kg (high dose), diluted with 0.9% NaCl to a concentration of 1.5 mL/kg (low dose), and 0.9% NaCl as vehicle were administered *i.p.* once daily for 6 days before zymosan injection (pre-treatment) for experimental setting (1) and *i.p.* 4 h and again 8 h after zymosan injection (post-induction) for experimental setting (2). At 4, 8, 24, 192 or 360 h after zymosan injection, mice were euthanized. The inflammatory exudate was obtained by washing the peritoneal cavity with 2 mL PBS. Cells in the exudates were immediately counted using Scepter 2.0 cell counter (Merck Millipore, Burlington, MA, USA) and labeled for subpopulation identification and quantification by Macs quant analyzer (Miltenyi Biotec, Bergisch Gladbach, Germany).

### 4.5. Flow Cytometry and Definition of the Resolution Index

Peritoneal exudates were centrifuged and suspended to obtain a suspension at 0.5 × 10^6^ cells/mL. The cells were labelled with the following antibodies: anti-CD45-VioBlue (clone REA737, Miltenyi); anti-F4/80-FITC (clone REA126, Miltenyi); anti-GR1-PE (clone RB6-8C5, Biolegend); anti-CD3-PE-Vio777 (clone REA606, Miltenyi); anti-CD19-PE-Vio777 (clone REA749, Miltenyi). CD45^+^ cells were sorted as follows: PMNs: GR1^+^, F4/80^−^; infiltrated monocytes: GR1^+^, F4/80^+^; resident macrophages: GR1^−^, F4/80^+,hi^; eosinophils: GR1^−^, F4/80^+, med^ and their specific SSC/FSC pattern; lymphocytes: GR1^−^, CD3^+^/CD19^+^. Counts for infiltrated monocytes and resident macrophages were combined to obtain the total monocyte/macrophage population. To quantify apoptotic PMNs, cells from peritoneal lavage fluid were stained with anti-CD45-VioBlue (Miltenyi), anti-F4/80-VioGreen (clone REA126, Miltenyi) and anti-Ly6G-PE (clone REA526, Miltenyi) antibodies. Apoptotic cells were labeled with anti-Annexin-V-FITC (Miltenyi) and DAPI (Miltenyi). This analysis allows to obtain the percentage of apoptotic PMNs among CD45^+^ cells and to calculate the cell number of this population. For efferocytosis experiments, cells were stained with anti-CD45-VioBlue (Miltenyi) and anti-F4/80-FITC (Miltenyi) antibodies to identify macrophages. Cells were then intracellularly stained with the anti-Ly6G-PE antibody (Miltenyi). This process allows quantifying the proportion of double-stained cells (Ly6G^+^, F4/80^+^) corresponding to apoptotic PMNs (Ly6G^+^) phagocyted by macrophages (F4/80^+^). Resolution indices were defined, including Ψ_max_ (maximal PMN counts), T_max_ (time point when PMNs reach Ψ_max_), Ψ_50_ (50% PMN number reduction), T_50_ (time point corresponding to Ψ_50_) and Ri (resolution interval, the interval between T_max_ and T_50_) according to [26].

### 4.6. Measurement of Lipid Mediators and Cytokines in Peritoneal Lavage

The extraction protocol and analysis of LM in peritoneal lavages was performed as previously described [25] and adapted according to the Ambiotis SAS (Toulouse, France) standard operating procedure. Dedicated samples of peritoneal lavages were extracted using oasis HLB 96 wells solid phase extraction (Waters Corporation, Milford, MA, USA). The LC-MS/MS analysis was performed on a 1290 Infinity UHPLC system coupled to a 6490 triple quadrupole MS (Agilent Technologies, Santa Clara, CA, USA), equipped with electrospray ionization source, and performed in negative ion mode. Reverse-phase UHPLC was performed with a Kinetex Biphenyl column (2.1 mm × 50 mm × 1.7 µm; Phenomenex Inc., Torrance, CA, USA) maintained at 50 °C. For monitoring and quantification of LM, analyses were run in multiple reaction monitoring (MRM) detection mode. Identification was conducted using pure authentic standards. Peak detection, integration, and quantitative analysis was done by use of MassHunter Quantitative Analysis Software version B.08.00 (Agilent Technologies, Santa Clara, CA, USA). Cytokines/chemokines (TNFα, IL-10, IL-6, KC (IL-8), eotaxin, and MCP-1) were analyzed in the peritoneal lavages with a 6-plex MILLIPLEX MAP Mouse Cytokine/Chemokine (Merck Millipore) according to manufacturer’s instructions. Analysis and quantification were performed by Luminex 100IS (Luminex, Austin, TX, USA). Ratio of IL-10/pro-inflammatory cytokines were calculated by dividing of the value of IL-10 through the sum of the values of IL-6, TNF-α, MCP-1, eotaxin-1 and KC (IL-8) of each animal.

### 4.7. Measurement of Gene Expression in the Peritoneal Lavage

mRNA was extracted from the peritoneal lavages using the TurboCapture 96 mRNA kit (Qiagen, Hilden, Germany). The reverse transcription was then carried out and the specific preparation steps for the chip (96 × 96) were conducted according to Fluidigm protocol version PN100-1201B1. In brief, a pre-amplification step was performed in the presence of the primers used on the chip using the Fluidigm Preamp Master Mix (Fluidigm, San Francisco, CA, USA). This pre-amplification was performed on a Veriti 96-well Fast Thermal Cycler (Applied Biosystems, Foster City, CA, USA) at 95 °C for 10 min followed by 14 cycles at 95 °C (15 s) and 60 °C (4 min). Each pre-amplified cDNA sample was diluted 1:5 and then deposited in a 96-well plate according to a pre-defined plate pan with a mix containing Taqman Gene expression Master Mix (Applied Biosystems), EvaGreen (Interchim, Montluçon, France) and Dye Sample Loading Reagent (Fluidigm). In parallel, on another 96-well plate, each pair of primer was prepared at a concentration of 5 µM according to a pre-defined plate plan. Then, each plate was deposited on both sides of the chip. The mixture of each well of each plate was made by the IFC controller within the chip and then the chip was placed in the BioMark (Fluidigm) to perform the RT-PCR. The data was extracted using Fluidigm Real-Time PCR analysis software (Fluidigm). The expression level of the mRNAs was normalized using the following reference genes: YWHAZ, OAZ1, HPRT and β-actin. Data were analyzed using the ΔΔCt method where the Ct corresponds to the number of cycles necessary to generate a fluorescent signal above the pre-defined threshold and with the cut-off criteria of Ct > 23. The results are shown as fold-increase against the mean of the vehicle group at each time point.

### 4.8. Isolation of Human Monocytes and Preparation of Monocyte-Derived Macrophages

Leukocyte concentrates from freshly withdrawn peripheral blood of healthy adult human donors were provided by the Institute of Transfusion Medicine (University Hospital Jena, Germany). The experimental protocol was approved by the ethical committee of the University Hospital Jena. All methods were performed in accordance with the relevant guidelines and regulations. PBMC were isolated using dextran sedimentation and Ficoll-Histopaque 1077-1 (Sigma-Aldrich, Taufkirchen, Germany) centrifugation. PBMC were seeded in PBS containing 1 mM Ca^2+^ and 0.5 mM Mg^2+^ in cell culture flasks (Greiner Bio-one, Frickenhausen, Germany) for 1.5 h at 37 °C and 5% CO_2_ for adherence of monocytes. For differentiation and polarization of monocytes towards M1- and M2-MDM, published criteria [27] were used. Thus, M1-MDM were generated by incubating monocytes with 20 ng/mL GM-CSF (Peprotech, Hamburg, Germany) for 6 days in RPMI 1640 supplemented with 10% fetal calf serum, 2 mmol/L L-glutamine (Biochrom/Merck, Berlin, Germany), and penicillin-streptomycin (Biochrom/Merck), followed by 100 ng/mL LPS (Sigma-Aldrich) and 20 ng/mL IFN-γ (Peprotech) treatment for another 48 h. M2-MDM were obtained after differentiation of monocytes with 20 ng/mL M-CSF (Peprotech) for 6 days, and then with 20 ng/mL IL-4 (Peprotech) for additional 48 h of polarization.

### 4.9. Bacterial Cultivation

Experiments with intact *Staphylococcus (S.) aureus* strains were performed as previously described [34]. In brief, bacteria were grown overnight at 37 °C in brain heart infusion (BHI) medium while shaking, diluted to optical density (OD) of 0.05 at 600 nm and grown for another 3 h (log-phase). Bacteria were washed in PBS and resuspended in PBS without Ca^2+^/Mg^2+^.

### 4.10. Cytotoxicity Analysis

The cytotoxicity of Tr14 was assessed by MTT assay. Unpolarized naïve MDM (1 × 10^5^ M_GM-CSF_ or M_M-CSF_) were seeded in a 96-well plate in the respective medium (100 µL/well). MDM were allowed to adhere for 1.5 h (37 °C, 5% CO_2_) before treatment. Tr14 at different dilutions (0.9% NaCl solution as vehicle) were added to each well and MDM were polarized with LPS/IFN-γ to M1 and with IL-4 to M2 at 37 °C for 48 h. Staurosporine (1 µM), a pan-kinase inhibitor and inducer of apoptosis, was used as positive control. Then, 20 µL of thiazolyl blue tetrazolium bromide (MTT, 5 mg/mL PBS) was added, and the incubation was continued at 37 °C and 5% CO_2_ until blue staining of the vehicle control. Formazan formation was stopped by adding 100 µL of lysis buffer (SDS, 10%, *w*/*v* in 20 mM HCl) and samples were shaken overnight. The absorbance of each well was measured at 570 nm in a Multiskan™ microplate spectrophotometer (Thermo Scientific, Ulm, Germany).

### 4.11. Incubation of Human MDM and LM Metabololipidomics

Unpolarized M_GM-CSF_ and M_M-CSF_ (2 × 10^6^) were pre-treated with 0.1 or 10% of Tr14 or vehicle (0.9% NaCl) for 15 min prior addition of the polarization agents (LPS plus IFN-γ for M1-MDM, and IL-4 for M2-MDM). After 48 h, cells were stimulated with *S. aureus* (LS1 strain) at a ratio of 1:50 (M1/M2-MDM: *S. aureus*) in PBS containing 1 mM CaCl_2_ for 180 min at 37 °C. Alternatively, polarized M1- and M2-MDM (2 × 10^6^, 48 h) were incubated in PBS containing 1 mM CaCl_2_ with 0.1 or 10% of Tr14 or vehicle (0.9% NaCl) and, after 15 min, *S. aureus* (LS1 strain) at a ratio of 1:50 (M1/M2-MDM: *S. aureus*) was added and incubation was prolonged for another 180 min at 37 °C. Supernatants of the above-mentioned incubations were transferred to 2 mL of ice-cold methanol containing 10 µL of deuterium-labeled internal standards (200 nM d8-5S-HETE, d4-LTB_4_, d5-LXA_4_, d5-RvD2, d4-PGE_2_ and 10 µM d8-AA) to facilitate quantification. Deuterated and non-deuterated LM standards were purchased from Cayman Chemical/Biomol GmbH (Hamburg, Germany). Sample preparation was conducted by adapting published criteria [27]. In brief, samples were kept at −20 °C for 60 min to allow protein precipitation. After centrifugation (1200 g, 4 °C, 10 min) 8 mL acidified water (pH 3.5) was added and subjected to solid-phase extraction using solid-phase cartridges (Sep-Pak^®^ Vac 6cc 500 mg/6 mL C18; Waters, Milford, MA). After washing with 6 mL water and additional 6 mL *n*-hexane, LMs were eluted with 6 mL methyl formate. Finally, the samples were brought to dryness using an evaporation system (TurboVap LV, Biotage, Uppsala, Sweden) and resuspended in 100 µL methanol-water (50/50, *v*/*v*) for UPLC-MS-MS automated injections. LM profiling was assessed with an Acquity™ UPLC system (Waters, Milford, MA) and a QTRAP 5500 Mass Spectrometer (ABSciex, Darmstadt, Germany) equipped with a Turbo V™ Source and electrospray ionization (ESI). LMs were eluted using an ACQUITY UPLC^®^ BEH C18 column (1.7 µm, 2.1 × 100 mm; Waters, Eschborn, Germany) at 50 °C with a flow rate of 0.3 mL/min and a mobile phase consisting of methanol-water-acetic acid of 42:58:0.01 (*v*/*v*/*v*) that was ramped to 86:14:0.01 (*v*/*v*/*v*) over 12.5 min and then to 98:2:0.01 (*v*/*v*/*v*) for 3 min [28]. The QTrap 5500 was operated in negative ionization mode using scheduled MRM coupled with information-dependent acquisition. The scheduled MRM window was 60 s, optimized LM parameters were adopted [39], and the curtain gas pressure was set to 35 psi. The retention time and at least six diagnostic ions for each LM were confirmed by means of an external standard assay (Cayman Chemical/Biomol GmbH, Hamburg, Germany). Quantification was achieved by calibration curves for each LM. Linear calibration curves were obtained for each LM and gave r^2^ values of 0.998 or higher (for fatty acids 0.95 or higher). Additionally, the limit of detection for each targeted LM was determined [28]. Following LM were analyzed: SPMs (PD1, PDX, RvD2, RvD5, MaR1, and LXA_4_), COX products (PGE_2_, PGD_2_, PGF_2α_ and TXB_2_), 5-LOX products (LTB_4_, trans- and epi-trans-LTB_4_ and 5-HETE), 15-LOX products (17-HDHA, 15-HEPE, and 15-HETE) and 12-LOX products (14-HDHA, 12-HEPE, and 12-HETE).

### 4.12. Quantification and Statistical Analysis

Results are expressed as mean ± standard error of the mean (SEM) of n observations, where n represents the number of experiments with separate donors, performed on different days, as indicated or mentioned otherwise. For the *in vivo* studies, n represents the number of animals. Analyses of data were conducted using GraphPad Prism 8 software (San Diego, CA, USA). Two-tailed t test was used for the comparison of two groups. For multiple comparisons, one-way and two-way analysis of variance (ANOVA) with Bonferroni, Dunnett´s and Tukey’s post hoc tests were applied as indicated. To identify outliers, ROUT test (Q = 0.1%) was performed and further outliers were excluded. The criterion for statistical significance is *p* < 0.05.

## 5. Conclusions

We demonstrated that Tr14 enhances the biosynthesis of 12-/15-LOX products and SPMs in zymosan-induced mouse peritonitis as well as in human MDM challenged with bacterial exotoxins. In the mouse peritonitis model, Tr14 also positively influenced the inflammation resolution index and supported efferocytosis. Our data reveal Tr14 as an immunoresolvent agent with diverse actions that promote resolution of inflammation, which may benefit patients with non-resolving inflammation due to defects in the resolution response.

## Data Availability

The data presented in this study are available on reasonable request from the corresponding author. The data are not publicly available due to privacy.

## Supplementary Materials

The following are available online at https://www.mdpi.com/article/10.3390/ph14111123/s1, Figure S1: Study plan for investigation of the effects of Tr14 on the resolution of inflammation in zymosan-induced mouse peritonitis, Figure S2: Self-resolving inflammation was initiated by injection of zymosan (0.1 mg/mouse, i.p.) into mice. Before zymosan injection, Tr14 (3 mL/kg or 1.5 mL/kg) or vehicle (veh., 0.9% NaCl) were administered i.p. once daily for six days. Peritoneal exudates were collected after 4, 8 and 24 h (*n* = 6–8; outliers were removed) post-zymosan injection, and from naïve untreated mice representing time point 0 h (*n* = 6). (A) Quantification of cell numbers of PMNs, eosinophils, monocytes/macrophages and lymphocytes in the peritoneum, shown as mean ± SEM for indicated time points, * *p* < 0.05; *** *p* < 0.001; *p* values were calculated versus vehicle for each time point; unpaired two-way ANOVA with Dunnett´s multiple comparison test. (B) Cell composition in the peritoneal exudates 4 h after zymosan injection against naïve untreated mice are shown in pie charts. (C) Cytokine levels were measured at indicated time points and shown in pg/mL exudate as mean ± SEM. (D) mRNA levels of inflammation-related genes in peritoneal lavages 4 h post-zymosan injection, analyzed by RT-PCR. Data are given as mean ± SEM as fold increase of vehicle group. (E) LM levels in peritoneal lavages (summarized in relevant groups: SPMs (LXA4, PD1, MaR1, RvD2 and RvD5), 12-LOX products (14-HDHA and 12-HETE), 15-LOX (17-HDHA and 15-HETE) and COX products (PGE2 and TXB2)) at the indicated time points were analyzed by UPLC-MS-MS and are shown in pg/mL exudate as mean ± SEM as line charts, Figure S3: Self-resolving inflammation was initiated by injecting zymosan (0.1 mg/mouse, i.p.) into mice. Tr14 (1.5 mL/kg or 3 mL/kg) or vehicle (veh., 0.9% NaCl) were administered i.p. 4 h and 8 h post-zymosan injection. Peritoneal exudates were collected 4, 8, 24, 192 and 360 h (*n* = 6–8; outliers were removed) post-zymosan injection, and from naïve untreated mice representing time point 0 h (*n* = 6). (A) Quantification of cell numbers of PMNs, eosinophils, lymphocytes, resident and recruited monocytes/macrophages as well as total monocytes/macrophages in the peritoneum, shown as mean ± SEM for the indicated time points; * *p* < 0.05; ** *p* < 0.01; *** *p* < 0.001; *p* values were calculated versus vehicle for each time point; unpaired two-way ANOVA with Dunnett´s multiple comparison test. (B) Cell composition in the peritoneal exudates 24 h and 360 h after zymosan injection against naïve untreated mice are shown in pie charts. (C) Cytokine levels were measured at indicated time points and shown in pg/mL exudate as mean ± SEM. (D) LM levels in peritoneal lavages (summarized in relevant groups: SPMs (LXA4, PD1, MaR1, RvD2 and RvD5), 12-LOX products (14-HDHA and 12-HETE), 15-LOX (17-HDHA and 15-HETE) and COX products (PGE2 and TXB2)) at the indicated time points were analyzed by UPLC-MS-MS and are shown in pg/mL exudate as mean ± SEM; ** *p* < 0.01; *p* values were calculated versus vehicle for each time point; unpaired two-way ANOVA with Dunnett´s multiple com-parison test. Figure S4: (A) Cytotoxicity of Tr14. Human naïve MDM (M_GM-CSF_ and M_M-CSF_, 1 × 10^5^) were incubated with different concentrations of Tr14 or 0.9% NaCl as vehicle or 1 µM staurosporine (Stsp.) as positive control and polarized for 48 h with LPS/IFNγ to M1- and with IL-4 to M2-MDM at 37 °C. M1- and M2-MDM were then incubated with MTT (5 mg/mL, 20 μL) for 2 h at 37 °C. The formazan product was solubilized with SDS (10% in 20 mM HCl) and the absorbance was measured at 570 nm. Results are given in % of control (0.9% NaCl), n = 3. (B) Human MDM were preincubated with 0.1 or 10% of Tr14 or vehicle (0.9% NaCl solution) for 15 min and then polarized for 48 h with IL-4 to M2-MDM. Then, cells were incubated with S. aureus (LS1; ratio 1:50) in PBS pH 7.4 containing 1 mM CaCl_2_ for another 180 min. Formed LM were extracted from the supernatants and analyzed by UPLC-MS-MS, and are given as means in pg/2 × 10^6^ cells ± SEM and % of vehicle is shown in a heat map; *n* = 3 separate donors. Table S1: Composition of Traumeel (Tr14), solution for injection.
