# Supplementary material for: The Natural Combination Medicine Traumeel (Tr14) Improves Resolution of Inflammation by Promoting the Biosynthesis of Specialized Pro-Resolving Mediators
Source: Pharmaceuticals (Basel). 2021 Nov 3;14(11):1123. doi: 10.3390/ph14111123 (PMC8623904; doi:10.3390/ph14111123)
Supplement: Supplementary file 1 [file pharmaceuticals-14-01123-s001.zip › pharmaceuticals-1435947-supplementary.pdf]

pharmaceuticals

Supplementary Materials

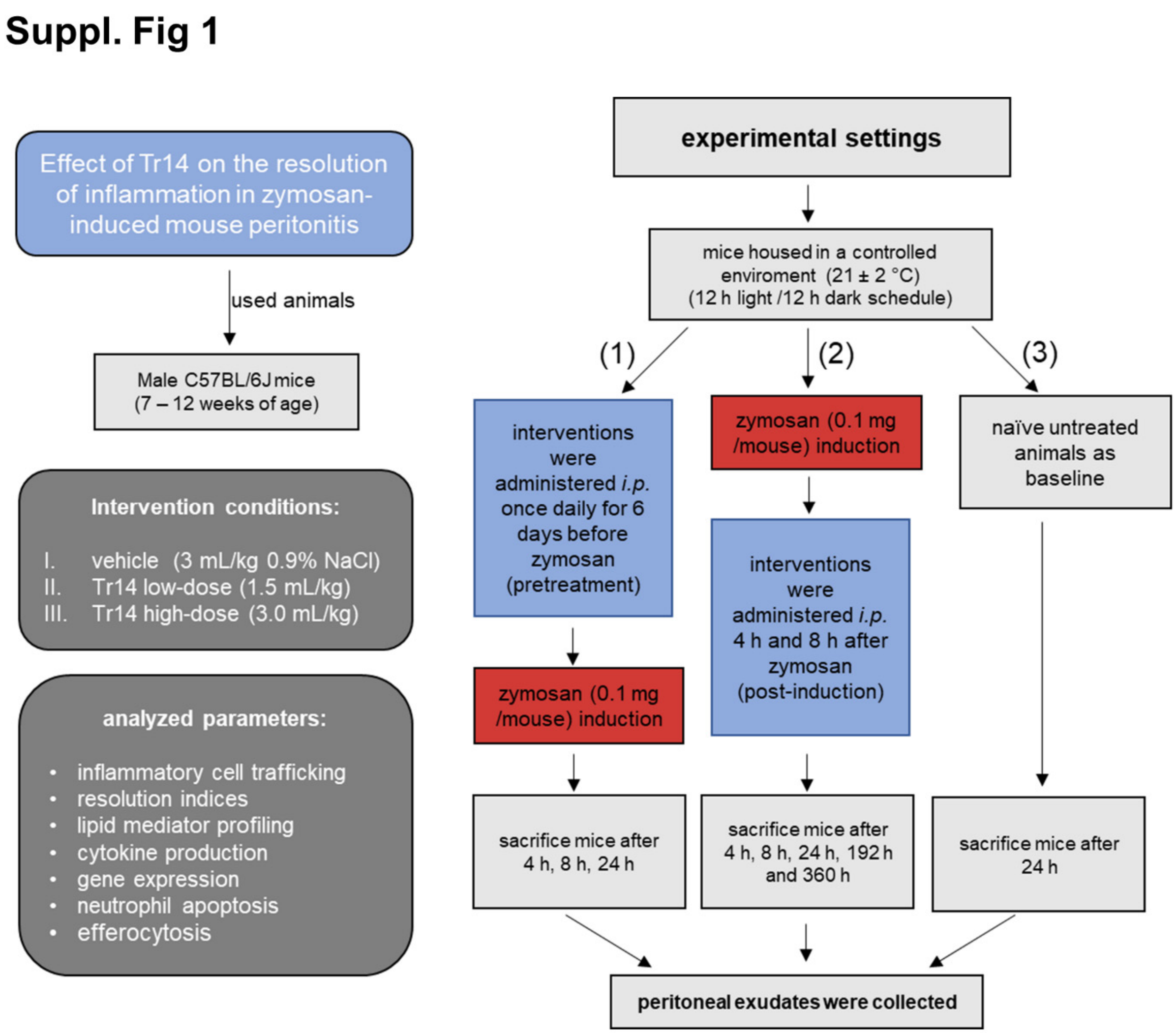

Supplemental Figure S1: Study plan for investigation of the effects of Tr14 on the resolution of inflammation in zymosan-induced mouse peritonitis.

Suppl. Fig 2

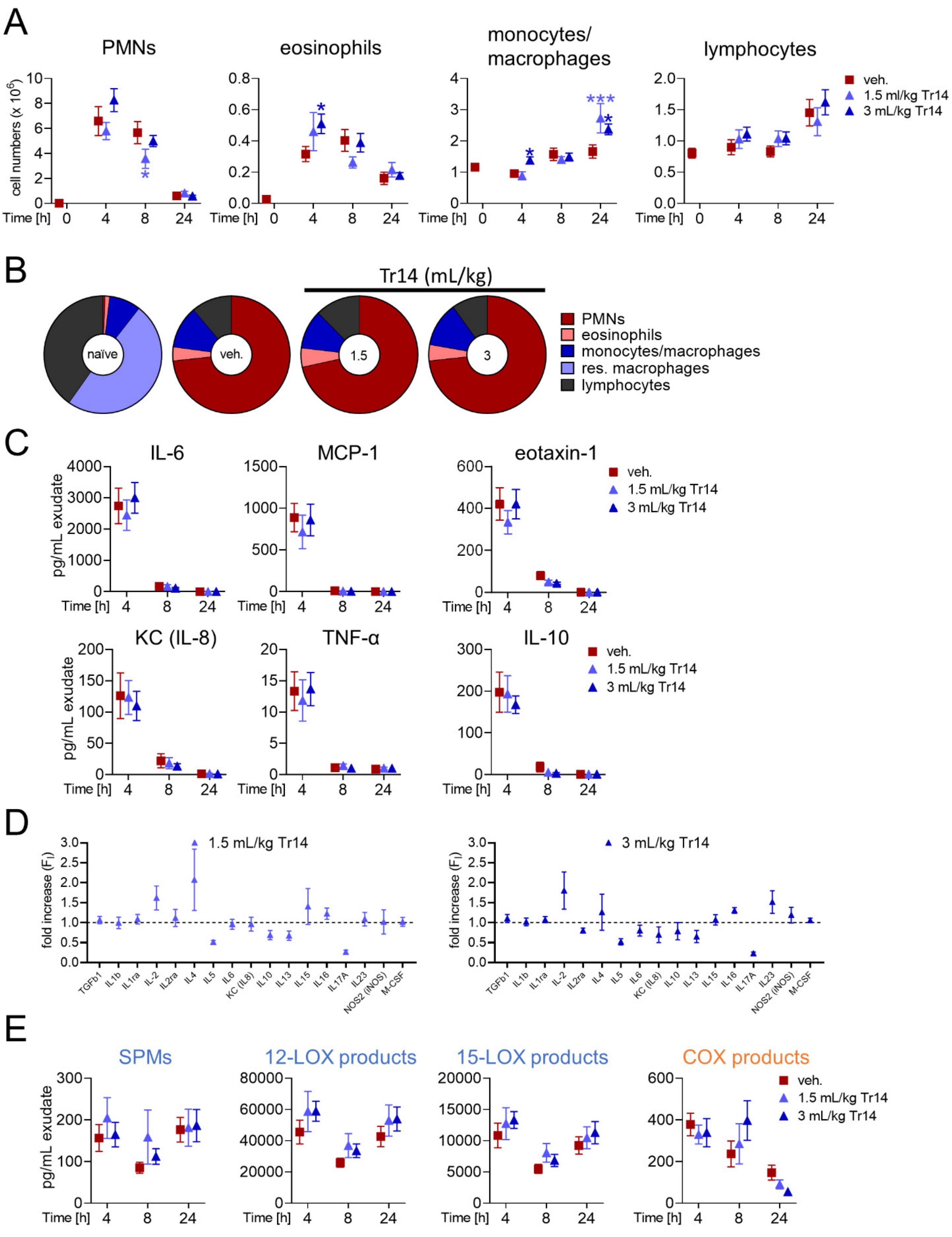
A
PMNs
eosinophils
monocytes/ macrophages
lymphocytes
cell numbers (x 10^6)
Time [h]
veh.
1.5 ml/kg Tr14
3 mL/kg Tr14
B
Tr14 (mL/kg)
naïve
veh.
1.5
3
PMNs
eosinophils
monocytes/macrophages
res. macrophages
lymphocytes
C
IL-6
MCP-1
eotaxin-1
KC (IL-8)
TNF-α
IL-10
pg/mL exudate
veh.
1.5 mL/kg Tr14
3 mL/kg Tr14
D
fold increase (Fi)
1.5 mL/kg Tr14
3 mL/kg Tr14
TGFb1
IL1b
IL1ra
IL-2
IL2ra
IL4
IL5
IL6
KC (IL8)
IL10
IL13
IL15
IL16
IL17A
IL23
NOS2 (iNOS)
M-CSF
E
SPMs
12-LOX products
15-LOX products
COX products
pg/mL exudate

Supplemental Figure S2: Self-resolving inflammation was initiated by injection of zymosan (0.1 mg/mouse, *i.p.*) into mice. Before zymosan injection, Tr14 (3 mL/kg or 1.5 mL/kg) or vehicle (veh., 0.9% NaCl) were administered *i.p.* once daily for six days. Peritoneal exudates were collected after 4, 8 and 24 h (n = 6-8; outliers were removed) post zymosan injection, and from naïve untreated mice representing time point 0 h (n = 6). (**A**) Quantification of cell numbers of PMNs, eosinophils, monocytes/macrophages and lymphocytes in the peritoneum, shown as mean ± SEM for indicated time points, * < p 0.05; ****p* < 0.001; *p* values were calculated versus vehicle for each time point; unpaired two-way ANOVA with Dunnett´s multiple comparison test. (**B**) Cell composition in the peritoneal exudates 4 h after zymosan injection against naïve untreated mice are shown in pie charts. (**C**) Cytokine levels were measured at indicated time points and shown in pg/mL exudate as mean ± SEM. (**D**) mRNA levels of inflammation-related genes in peritoneal lavages 4 h post zymosan injection, analyzed by RT-PCR. Data were given as mean ± SEM as fold increase of vehicle group. (**E**) LM levels in peritoneal lavages (summarized in relevant groups: SPMs (LXA_4, PD1, MaR1, RvD2 and RvD5), 12-LOX products (14-HDHA and 12-HETE), 15-LOX (17-HDHA and 15-HETE) and COX products (PGE_2 and TXB_2)) at the indicated time points were analyzed by UPLC-MS-MS and are shown in pg/mL exudate as mean ± SEM as line charts.

Suppl. Fig 3

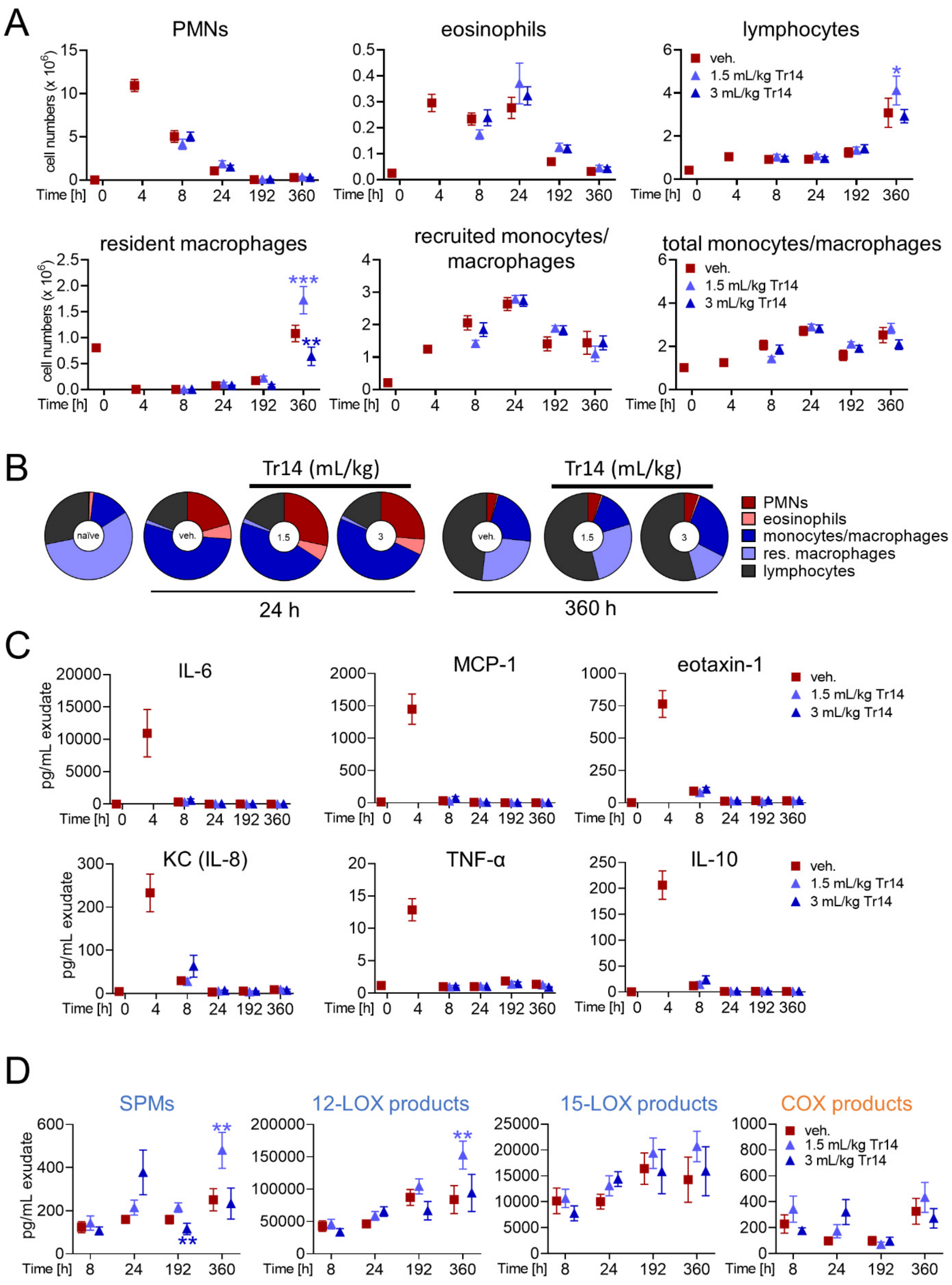
A
PMNs
eosinophils
lymphocytes
cell numbers (x 10^6)
Time [h]
veh.
1.5 mL/kg Tr14
3 mL/kg Tr14
resident macrophages
recruited monocytes/ macrophages
total monocytes/macrophages
B
Tr14 (mL/kg)
naïve
veh.
1.5
3
24 h
360 h
PMNs
eosinophils
monocytes/macrophages
res. macrophages
lymphocytes
C
IL-6
MCP-1
eotaxin-1
pg/mL exudate
KC (IL-8)
TNF-α
IL-10
D
SPMs
12-LOX products
15-LOX products
COX products

Supplemental Figure S3: Self-resolving inflammation was initiated by injecting zymosan (0.1 mg/mouse, *i.p.*) into mice. Tr14 (1.5 mL/kg or 3 mL/kg) or vehicle (veh., 0.9% NaCl) were administered *i.p.* 4 h and 8 h post zymosan injection. Peritoneal exudates were collected 4, 8, 24, 192 and 360 h (n = 6-8; outliers were removed) post zymosan injection, and from naïve untreated mice representing time point 0 h (n = 6). (**A**) Quantification of cell numbers of PMNs, eosinophils, lymphocytes, resident and recruited monocytes/ macrophages as well as total monocytes/macrophages in the peritoneum, shown as mean ± SEM for the indicated time points; $^{*}p < 0.05$; $^{**}p < 0.01$; $^{***}p < 0.001$; *p* values were calculated versus vehicle for each time point; unpaired two-way ANOVA with Dunnett´s multiple comparison test. (**B**) Cell composition in the peritoneal exudates 24 h and 360 h after zymosan injection against naïve untreated mice are shown in pie charts. (**C**) Cytokine levels were measured at indicated time points and shown in pg/mL exudate as mean ± SEM. (**D**) LM levels in peritoneal lavages (summarized in relevant groups: SPMs (LXA_4, PD1, MaR1, RvD2 and RvD5), 12-LOX products (14-HDHA and 12-HETE), 15-LOX (17-HDHA and 15-HETE) and COX products (PGE_2 and TXB_2)) at the indicated time points were analyzed by UPLC-MS-MS and are shown in pg/mL exudate as mean ± SEM; $^{**}p < 0.01$; *p* values were calculated versus vehicle for each time point; unpaired two-way ANOVA with Dunnett´s multiple comparison test.

Suppl. Fig 4

A

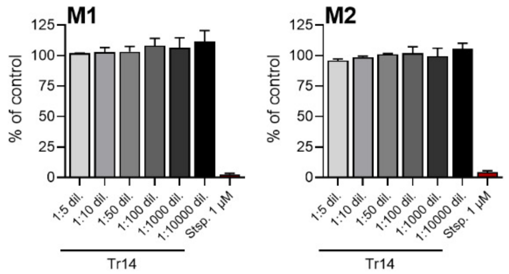

B

0 100 500
%

		veh.			Tr14 (0.1%)			%	Tr14 (10%)			%
bioactive SPM	RvD5	486	±	262	673	±	443	139	1,014	±	532	209
	RvD2	8.4	±	2.7	11	±	4.2	129	17	±	6.0	198
	RvD1	13	±	3.3	17	±	4.7	133	24	±	5.8	192
	MaR1	42	±	15	64	±	38	153	88	±	36	208
	MaR2	43	±	12	59	±	29	136	78	±	47	180
	PD1	9.9	±	2.7	14	±	4.9	137	17	±	5.1	171
	PDX	25	±	9.3	36	±	19	144	48	±	19	194
	LXA_4	26	±	12	30	±	3.3	113	30	±	3.3	113
	5,15-diHETE	425	±	122	540	±	218	127	776	±	245	182
SPM precursors	18-HEPE	127	±	32	153	±	35	120	180	±	13	142
	17-HDHA	17,390	±	5,110	23,016	±	9,928	132	27,904	±	10,411	160
	15-HEPE	2,796	±	534	3,501	±	1,136	125	4,482	±	1,011	160
	15-HETE	21,009	±	2,129	24,645	±	5,792	117	30,440	±	5,106	145
	14-HDHA	1,505	±	484	1,891	±	810	126	2,768	±	870	184
	12-HEPE	323	±	76	386	±	129.5	119	540	±	130	167
	12-HETE	1,084	±	219	1,289	±	360	119	1,877	±	310	173
	11-HEPE	89	±	20	107	±	27	120	121	±	17	135
	11-HETE	812	±	185	935	±	231	115	1,147	±	178	141
	10-HDHA	646	±	190	794	±	336	123	1,151	±	359	178
	8-HETE	433	±	58	531	±	119	123	685	±	128	158
	7-HDHA	299	±	35	409	±	118	137	481	±	107	161
	4-HDHA	150	±	40	183	±	27	122	213	±	19	142
5-LOX	LTB_4	93	±	31	109	±	26	116	109	±	9.0	117
	t-LTB_4	53	±	13	56	±	3.8	107	69	±	2.7	131
	20-OH-LTB4	0.6	±	0.1	0.8	±	0.3	144	0.9	±	0.3	155
	5-HETE	1,055	±	363	1,232	±	273	117	1,299	±	227	123
	5-HEPE	200	±	77	243	±	77	122	253	±	57	127
COX	PGE_2	185	±	66	208	±	39	112	240	±	15	129
	PGD_2	65	±	23	72	±	19	110	86	±	16	132
	$PGF_{2\alpha}$	952	±	417	1,044	±	317	110	1,094	±	235	115
	TXB_2	19,772	±	7,319	23,869	±	6,969	121	25,169	±	6,242	127
fatty acids	AA	1,689,082	±	415,484	1,685,400	±	255,420	100	1,681,649	±	263,883	100
	EPA	632,025	±	160,029	642,925	±	100,045	102	648,950	±	114,521	103
	DHA	320,482	±	71,164	335,587	±	41,132	105	330,627	±	47,100	103

Supplemental Figure S4: (**A**) Cytotoxicity of Tr14. Human naïve MDM (M_{GM-CSF} and M_{M-CSF}, 1×10^5) were incubated with different concentrations of Tr14 or 0.9% NaCl as vehicle or 1 µM staurosporine (Stsp.) as positive control and polarized for 48 h with LPS/IFNγ to M1- and with IL-4 to M2-MDM at 37 °C. M1- and M2-MDM were then incubated with MTT (5 mg/mL, 20 µL) for 2 h at 37 °C. The formazan product was solubilized with SDS (10% in 20 mM HCl) and the absorbance was measured at 570 nm. Results are given in % of control (0.9% NaCl), n = 3. (**B**) Human MDM were preincubated with 0.1 or 10% of Tr14 or vehicle (0.9% NaCl solution) for 15 min and then polarized for 48 h with IL-4 to M2-MDM. Then, cells were incubated with *S. aureus* (LS1; ratio 1:50) in PBS pH 7.4 containing 1 mM $CaCl_2$ for another 180 min. Formed LM were extracted from the supernatants and analyzed by UPLC-MS-MS, and are given as means in pg/2×10^6 cells ± SEM and % of vehicle was shown in a heat map; n = 3 separate donors.

Supplemental Table S1: Composition of Traumeel (Tr14), solution for injection.

Component	Manufacturing method (Ph. Eur.)	µg/mL in Tr14
Plant extracts		
Achillea millefolium L. (common yarrow), fresh aerial parts collected at flowering time.	Method 1.1.5	3
Aconitum napellus L. (monkshood), fresh whole plants collected at the start of flowering.	Method 1.1.3	12
Arnica montana L. (arnica), dried underground parts.	Method 1.1.8	100
Atropa belladonna L. (deadly nightshade), whole fresh flowering plant harvested at the end of flowering, with the ligneous base of the stems removed.	Method 1.1.3	20
Bellis perennis L. (daisy), whole fresh flowering plants.	Method 1.1.3	10
Calendula officinalis L. (pot marigold), fresh aerial parts collected at flowering time.	Method 1.1.5	30
Echinacea; whole fresh flowering plants of *Echinacea angustifolia* D.C. (narrow-leaf purple coneflower) and *Echinacea pallida* (Nutt.) Nutt. (pale purple coneflower), single species or mixed.	Method 1.1.5	7.5
Echinacea purpurea (L.) Moench (Eastern purple coneflower), fresh aerial parts collected at flowering time.	Method 1.1.5	7.5
Hamamelis virginiana L. (American witch-hazel), fresh bark from roots or branches or a mixture thereof.	Method 1.1.5	30
Hypericum perforatum L (common St. John's-wort), whole fresh plant collected at the beginning of the flowering period.	Method 1.1.5	9
Matricaria recutita L. (German chamomile), whole fresh flowering plants.	Method 1.1.5	3
Symphytum officinale L. (common comfrey), fresh underground parts collected prior to flowering.	Method 1.1.5	0.003
Chemical substances		
Hepar sulfuris, obtained by calcining a mixture of Calcium carbonicum Hahnemanni and sulfur.	Method 4.1.1	0.001
Mercurius solubilis Hahnemanni, a mixture of mainly mercury (II) amidonitrate and metallic mercury.	Method 4.1.1	0.0005
